# Clinical features and survival analysis of cutaneous metastases from visceral cancers: A multicenter retrospective study

**DOI:** 10.1016/j.jdin.2026.04.018

**Published:** 2026-05-05

**Authors:** Pablo Balado-Simó, Xavier Fustà-Novell, Ana Talavera-Belmonte, Montserrat Bonfill-Ortí, Xavier Cubiró, Lluís Puig, Carlos González-Cruz, Vicente García-Patos, Álvaro March-Rodríguez, Ramon M. Pujol, Nina Richarz, Carlos Ferrándiz, Camila Cortés-Pinto, Xavier Soria-Gili, Amalia Luna, Sebastian Podlipnik

**Affiliations:** a Servicio de Dermatología, Hospital Clínic de Barcelona, Universitat de Barcelona, Barcelona, Spain; b Servicio de Dermatología, Hospital Universitari de Bellvitge, L'Hospitalet de Llobregat, Barcelona, Spain; c Servicio de Dermatología, Hospital de la Santa Creu i Sant Pau, Universitat Autònoma de Barcelona, Barcelona, Spain; d Servicio de Dermatología, Hospital Universitari Vall d'Hebron, Barcelona, Spain; e Servicio de Dermatología, Hospital del Mar, Barcelona, Spain; f Servicio de Dermatología, Hospital Universitari Germans Trias i Pujol, Badalona, Spain; g Servicio de Dermatología, Hospital Universitari Arnau de Vilanova, LLeida, Spain; h Melanoma Group, Institut d'Investigacions Biomèdiques August Pi i Sunyer, Barcelona, Spain

**Keywords:** breast neoplasms, cutaneous metastases, lung neoplasms, prognostic factors, survival analysis, visceral malignancies

*To the Editor:* Cutaneous metastases from visceral malignancies (CMVM) are uncommon manifestations of internal cancers but represent an important diagnostic and prognostic event. They occur in approximately 5% of patients with internal malignancies and often indicate advanced disease.^1^ Their clinical presentation is heterogeneous and may mimic several dermatologic conditions, delaying recognition.^2^ Survival outcomes vary depending on tumur type and disease burden, yet most studies include small cohorts or single-center designs.^3^

We conducted a retrospective multicenter cohort study across 7 tertiary hospitals in Catalonia, Spain, including patients with histologically confirmed CMVM diagnosed between April 2006 and October 2023, with follow-up until July 2025. Cases due to direct tumor extension, iatrogenic implantation, hematologic malignancies, or metastases from primary skin cancers were excluded. Demographic, clinical, and histopathologic variables were retrieved from electronic medical records. Overall survival was analyzed using Kaplan–Meier methods and multivariable Cox proportional hazards regression (details in Supplementary Material, available via Mendeley at https://data.mendeley.com/datasets/tfstjk5nks/1).

A total of 379 patients were included (219 women and 160 men; median age 65 years) (Supplementary Table I, available via Mendeley at https://data.mendeley.com/datasets/tfstjk5nks/1). Breast carcinoma was the most frequent primary tumor in women (61.6%), while lung carcinoma predominated in men (38.1%), consistent with previous reports.^1^^,^^2^ In 23.1% of cases, the cutaneous metastasis represented the first manifestation of an underlying malignancy.^1^ Lesions most frequently involved the trunk (57%) and presented as nodules or tumors (75.2%). Approximately half of patients had multiple lesions, and visceral metastases were present in 68.3%.

Survival differed markedly according to the primary tumor type as illustrated in the Kaplan–Meier survival curves (Fig 1). In multivariable analysis (Table I), lung cancer (HR 3.76, 95% CI 2.27-6.25), colorectal cancer (HR 2.54, 95% CI 1.24-5.09), and upper airway tumors (HR 6.28, 95% CI 3.06-12.95) were independently associated with increased mortality compared with breast carcinoma. The presence of visceral metastases also significantly worsened prognosis, both when previously known (HR 2.80, 95% CI 1.82-4.30) and when newly detected at CMVM diagnosis (HR 1.84, 95% CI 1.18-2.87). Conversely, multiple cutaneous lesions (HR 0.73, 95% CI 0.54-0.99) and unknown primary tumor at presentation (HR 0.62, 95% CI 0.41-0.96) were associated with improved survival (extended results are provided in the Supplementary Material, available via Mendeley at https://data.mendeley.com/datasets/tfstjk5nks/1).

**Fig 1:**
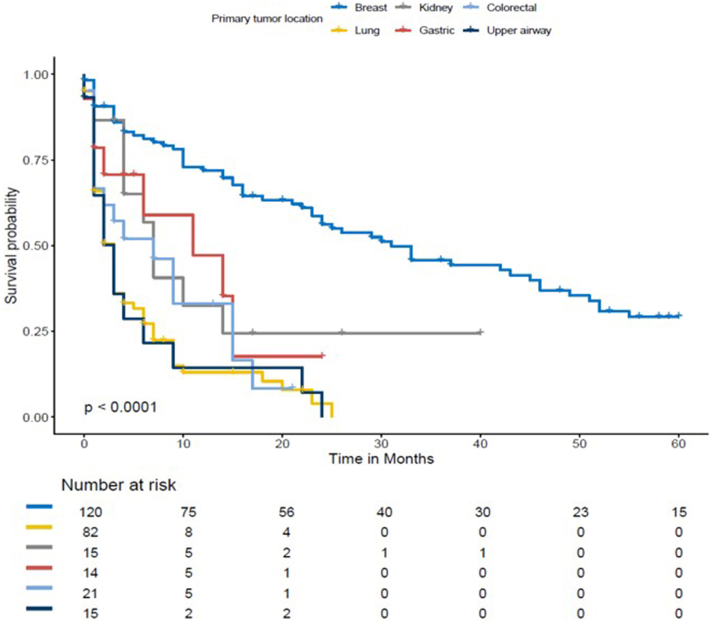
Kaplan–Meier curve for overall survival. Kaplan–Meier curves depicting overall survival stratified by primary tumor type. The risk table displays the number of patients at risk at each time point. Statistical significance was assessed using the log-rank test.

**Table I:** Univariable and multivariable Cox regression analysis for overall survival

	*n* (%)	HR (Univariable)	HR (Multivariable)
Age			
≥65	197 (52.0)	Reference	-
<65	182 (48.0)	0.93 (0.71-1.21, *P* = .570)	-
Sex			
Female	219 (57.8)	Reference	Reference
Male	160 (42.2)	2.37 (1.80-3.12, *P* < .001)	1.30 (0.86-1.99, *P* = .216)
Primary tumor location			
Breast	157 (46.3)	Reference	Reference
Lung	90 (30.4)	5.95 (3.98-8.91, *P* < .001)	3.76 (2.27-6.25, *P* < .001)
Kidney	15 (5.1)	2.47 (1.25-4.90, *P* = .009)	1.70 (0.80-3.67, *P* = .168)
Gastric	15 (5.1)	2.74 (1.28-5.85, *P* = .009)	1.84 (0.81-4.19, *P* = .148)
Colorectal	23 (7.8)	4.04 (2.26-7.25, *P* < .001)	2.54 (1.24-5.09, *P* = .007)
Upper airway	16 (5.4)	6.84 (3.40-13.76, *P* < .001)	6.28 (3.06-12.95, *P* < .001)
Number of lesions			
Single	180 (47.6)	Reference	Reference
Multiple	198 (52.4)	0.77 (0.59-1.00, *P* = .051)	0.73 (0.54-0.99, *P* = .041)
Visceral metastasis			
Absent	121 (33.7)	Reference	Reference
Yes, previously known	142 (37.6)	2.47 (1.77-3.45, *P* < .001)	2.80 (1.82-4.30, *P* < .001)
Yes, previously unknown	115 (30.4)	1.94 (1.40-2.79, *P* < .001)	1.84 (1.18-2.87, *P* = .007)
Known primary tumor			
Yes	293 (77.3)	Reference	Reference
No	86 (22.7)	0.59 (0.51-0.92, *P* = .013)	0.62 (0.41-0.96, *P* = .032)
Lesion type			
Tumor or nodule	285 (76.2)	Reference	Reference
Papule	35 (9.4)	0.91 (0.56-1.47, *P* = .692)	-
Plaque	54 (14.4)	0.63 (0.41-0.96, *P* = .032)	-

*CI*, Confidence interval; *HR*, hazard ratio.

This multicenter study represents, to our knowledge, the largest cohort specifically analyzing survival in patients with CMVM. Our results confirm that the biological behavior of the primary tumor is the main determinant of prognosis. Breast cancer showed the most favorable survival, whereas lung tumors were associated with poorer outcomes, consistent with previous reports.^4^ The apparently protective association of multiple cutaneous lesions may reflect slower-growing tumors, particularly hormone-driven malignancies such as breast carcinoma.^3^

CMVM remain a diagnostic challenge because their presentation often mimics benign or inflammatory dermatoses.^2^ Metastasis was clinically suspected in only a minority of cases in our cohort. Because CMVM may represent the first manifestation of an internal malignancy, prompt biopsy of atypical or rapidly growing lesions remains essential.^5^

Despite the retrospective design and the absence of detailed treatment data, this large multicenter cohort provides evidence regarding prognostic factors in CMVM and highlights the relevance of dermatologic evaluation in suspected metastatic disease.

## Conflicts of interest

None disclosed.
